# Human coronavirus dependency on host heat shock protein 90 reveals an antiviral target

**DOI:** 10.1080/22221751.2020.1850183

**Published:** 2020-12-17

**Authors:** Cun Li, Hin Chu, Xiaojuan Liu, Man Chun Chiu, Xiaoyu Zhao, Dong Wang, Yuxuan Wei, Yuxin Hou, Huiping Shuai, Jianpiao Cai, Jasper Fuk-Woo Chan, Jie Zhou, Kwok Yung Yuen

**Affiliations:** a State Key Laboratory of Emerging Infectious Diseases, The University of Hong Kong, Hong Kong, People’s Republic of China; b Department of Microbiology, The University of Hong Kong, Hong Kong, People’s Republic of China; c Carol Yu Centre for Infection, The University of Hong Kong, Hong Kong, People’s Republic of China

**Keywords:** Coronavirus, Hsp90β, SARS-CoV-2, nucleoprotein, viral replication

## Abstract

Rapid accumulation of viral proteins in host cells render viruses highly dependent on cellular chaperones including heat shock protein 90 (Hsp90). Three highly pathogenic human coronaviruses, including MERS-CoV, SARS-CoV and SARS-CoV-2, have emerged in the past 2 decades. However, there is no approved antiviral agent against these coronaviruses. We inspected the role of Hsp90 for coronavirus propagation. First, an Hsp90 inhibitor, 17-AAG, significantly suppressed MERS-CoV propagation in cell lines and physiological-relevant human intestinal organoids. Second, siRNA depletion of Hsp90β, but not Hsp90α, significantly restricted MERS-CoV replication and abolished virus spread. Third, Hsp90β interaction with MERS-CoV nucleoprotein (NP) was revealed in a co-immunoprecipitation assay. Hsp90β is required to maintain NP stability. Fourth, 17-AAG substantially inhibited the propagation of SARS-CoV and SARS-CoV-2. Collectively, Hsp90 is a host dependency factor for human coronavirus MERS-CoV, SARS-CoV and SARS-COV-2. Hsp90 inhibitors can be repurposed as a potent and broad-spectrum antiviral against human coronaviruses.

## Introduction

Coronaviruses are enveloped non-segmented positive-sense RNA viruses and infect various avian populations, bats, and mammals including humans [1]. In the past two decades, three highly pathogenic human coronaviruses have emerged, including severe acute respiratory syndrome coronavirus (SARS-CoV), Middle East respiratory syndrome coronavirus (MERS-CoV) and the current SARS-CoV-2 [2,3]. SARS-CoV resulted in the SARS outbreak in 2002–2003, affecting more than 8000 individuals globally with a case fatality rate of around 10% [4]. MERS-CoV emerged in the Middle East in 2012 [5], and has caused over 2500 laboratory-confirmed infection cases by November 2020, with an approximate mortality rate of 35%. COVID-19 caused by SARS-CoV-2 is the first coronavirus pandemic in history. To date, the morbidity and mortality of COVID-19 have reached an epic magnitude. These three coronaviruses primarily manifest as a respiratory infection, ranging from the upper respiratory infection to viral pneumonia leading to fatal outcome. Currently, there is no approved antiviral agents and vaccines for the treatment and prevention of human coronaviruses.

Coronaviruses have a large single-stranded RNA genome; two-thirds of the viral genome is translated into two polyproteins, and subsequently cleaved by two proteases into 15–16 non-structural proteins. The remaining one-third of the viral genome is transcribed into 9 subgenomic RNAs and encodes four structural proteins, spike, envelope, membrane and nucleocapsid as well as several accessory proteins [6]. Similar to other obligate pathogens, human coronaviruses rely on cellular machinery for viral infection and propagation. MERS-CoV utilizes cellular peptidase, dipeptidyl peptidase 4 (DPP4), for cellular entry [7]. Angiotensin-converting enzyme 2 (ACE2) has been identified to be the receptor for SARS-CoV and SARS-CoV-2 [8,9]. We and others have identified a number of attachment factors and co-receptors for MERS-CoV, including carcinoembryonic antigen-related cell adhesion molecule 5 (CEACAM5) and 78-kDa glucose-regulated protein (GRP78) [10,11].

Heat shock protein 90 (Hsp90) protein family represents one of the most abundantly expressed molecular chaperones and are highly conserved from bacteria to eukaryotes. Hsp90s contain three domains, N-terminal ATPase domain for ATP binding, middle domain for client protein binding and C-terminal domain for dimerization [12]. In mammalian cells, there are two cytosolic isoforms of Hsp90, the stress-inducible Hsp90α and constitutively expressed Hsp90β. These chaperone molecules play important roles in cellular protein homeostasis, including protein folding, protein maturation and degradation [13]. Apart from multiple functions of Hsp90 in proteostasis, many viruses are highly dependent on these cellular chaperones since rapidly-synthesized viral proteins require these chaperons for their proper folding and function. Hsp90 is essential for the maturation of the viral capsid proteins during viral replication of picornaviruses and vaccinia virus [14,15]. Hsp90 can facilitate the folding of viral structural proteins among several different viruses [16]. Overall, Hsp90 protein family are required for the replication of numerous viruses, including DNA viruses, double-stranded RNA viruses, positive- and negative- stranded RNA viruses [17].

There is a growing recognition of targeting virus dependency factor as an antiviral strategy, since host targeting has few concerns of evolving drug-resistant virus variants. Moreover, the cellular chaperon machinery has evolved a progressive diversification of isoforms and cofactors [18]. Viruses depend on a subset of cellular chaperons for replication, whereas host cells distribute the burden of proteostasis across the diversified chaperone network. As such, this provides a therapeutic opportunity for temporary manipulation of certain chaperons to inhibit virus replication with doses and duration tolerable in uninfected normal cells. The current dilemma of lacking effective antiviral agents for human coronaviruses prompted us to identify the host dependency factors of these life-threatening viruses. In this study, the initial observation of substantially-compromised MERS-CoV replication upon pharmacological inhibition of Hsp90 incited us to perform a series of in vitro studies to dissect the essential role of Hsp90 for the propagation of MERS-CoV, SARS-CoV and SARS-CoV-2. Overall, our study identified Hsp90 as an essential host determinant for human coronavirus replication, indicating Hsp90 as a potential treatment target against human coronavirus infections.

## Materials and methods

### Cell and virus culture

293T, Huh7 and A549 cells were maintained in Dulbecco’s Modified Eagle’s Medium (DMEM, Life Technologies) supplemented with 10% fetal bovine serum (FBS, Life Technologies), 100 IU/ml penicillin and 100 µg/ml streptomycin (P/S). Human embryonic lung fibroblast (HELF) cells were cultured in Minimum Essential Media (MEM, Life Technologies) with 20% FBS and P/S. MERS-CoV, SARS-CoV (GZ50, GenBank accession number AY304495) and SARS-CoV-2 (GenBank accession number MT230904) were propagated in Vero-E6 cells (ATCC) with serum-free DMEM. Two or three days after virus inoculation, cell-free culture media were harvested, titrated in Vero-E6 cells by plaque assay as we described elsewhere [19] and then stored in −80°C freezer in aliquots.

### Viral infection in cells and small intestinal organoids

After pre-treated with an Hsp90 inhibitor 17-allylamino-17-demethoxygeldanamycin (17-AAG, Tocris) for 1 h, cells and differentiated human small intestinal organoids established previously [20] were inoculated with the indicated coronaviruses and incubated in the presence of 17-AAG or DMSO. At the indicated hours post infection, virus-infected cells or organoids were harvested and applied to RNA extraction using MiniBEST Universal RNA Extraction kit (Takara). Cell-free culture media were harvested for RNA extraction using the MiniBEST Viral RNA/DNA Extraction Kit (Takara). Virus-specific primers were used in reverse transcription to generate complementary DNAs (cDNAs) for viral RNA. qPCR assay was performed with LightCycler 480 SYBR Green I Master Mix (Roche) to detect viral gene copy as we described previously [21]. Viral gene copy of SARS-CoV-2 was determined by one-step RT-qPCR assay (QuantiNova Probe RT-PCR kit, Qiagen) as described previously [20]. Human small intestinal organoids are maintained and differentiated according to our protocol described elsewhere [20]. The differentiated enteroids were mechanically sheared with and incubated with MERS-CoV at an estimated multiplicity of infection (MOI) of 0.1 at 37°C for 2 h. After washing, the inoculated enteroids were re-embedded in Matrigel (Corning) incubated with culture medium.

### Genetic depletion of Hsp90 with siRNA and subsequent experiments

A549 cells in a 24-well plate were transfected with Hsp90α siRNA (siHsp90α), Hsp90β siRNA (siHsp90β) or scrambled siRNAs (siSCR, Dharmacon) three times with a final concentration of 75 nM using Lipofectamine RNAiMAX (Life Technologies). At 24 h after the third transfection, the transfected cells were inoculated with MERS-CoV at a MOI of 0.1. The protein level of Hsp90α and Hsp90β in transfected cells was analysed by Western blot. Briefly, the cells were harvested with RIPA buffer, separated in 12% SDS-PAGE and then transferred to 0.22 µm PVDF membrane (Bio-Rad). After overnight blocking with 5% skim milk (Bio-Rad), the membrane was incubated with an anti-Hsp90α or anti-Hsp90β antibody (Thermo Fisher Scientific, PA3-013, PA3-012) for 2 h at room temperature, followed by incubation with HRP-conjugated anti-Rabbit antibodies and detection with immobilon crescendo western HRP substrate (Millipore). The infected cells were also fixed and immunolabelled for flow cytometry analysis to determine the infection rate as described elsewhere [22]. Briefly, the cells were detached with 10 mM EDTA in PBS, fixed in 4% PFA, permeabilized with 0.1% Triton X-100 in PBS, followed by immunolabeling with an in-house-made antibody against MERS-CoV NP and applied to flow cytometry using a FACSCanto II flow cytometer (BD Biosciences). Data were analysed using FlowJo version X (Tree Star).

At 24 h after twice transfection of siHsp90α, siHsp90β or siSCR, A549 cells were co-transfected with 2 µg NP plasmid and respective siRNAs with Lipofectamine™ 3000 and incubated for 48 h, followed with Western blot to detect NP protein. Alternatively, at 24 h after twice transfection with siHsp90β or siSCR, A549 cells were co-transfected with NP plasmid and siHsp90β or siSCR. At 5 h post co-transfection, the cells were incubated in presence or absence of 1.25 µM proteasome inhibitor MG132 (Tocris) for 43 h, and then harvested for Western blot to detect NP protein.

### Co-immunoprecipitation assay

pCMV3-flag-Hsp90β expressing an N-terminal FLAG-tagged Hsp90β was purchased from Sino Biological (HG11381-NF). Four pCAGEN-His plasmids were constructed, expressing four MERS-CoV structural proteins including envelope, membrane, nucleocapsid and spike. 293T cells seeded in a 10-cm dish were co-transfected with pCMV3-flag-Hsp90β and one of the four plasmids or blank vector using lipofectamine 3000 (Life Technologies). At 48 h post transfection, co-immunoprecipitation (Co-IP) assay was performed with Pierce^TM^ Classic Magnetic IP/Co-IP Kit (Thermo scientific, 88804). In brief, the transfected cells were lysed with ice-cold IP lysis/wash buffer supplemented with phosphatase inhibitor cocktails (Roche). After centrifugation, an aliquot of 500 µl supernatant was incubated with 2 µg of anti-Hsp90β antibody (ThermoFisher, PA3-012) overnight at 4°C, followed by incubation with 25 µl of pre-washed pierce protein A/G magnetic beads at room temperature for 1 h with mixing. After washing, the beads were eluted with 100 μl of elution buffer. The eluents were separated in 12% SDS-PAGE and then applied to Western blot analysis.

### Immunofluorescence staining

Virus-infected or mock-infected intestinal organoids, after fixation with 4% PFA, permeabilization with 0.5% Triton X-100, blocking with protein block buffer (Dako), were applied to immunofluorescence staining using an in-house-made antibody against MERS-CoV NP raised in guinea pig and a secondary antibody, goat-anti-guinea pig IgG Alexa Fluor 488 (A-11034, Invitrogen). Nuclei and actin filaments were counterstained with DAPI (Thermo Fisher Scientific) and Phalloidin-647 (Sigma-Aldrich), respectively. The organoids were then whole mounted on glass slide with ProLong™ Glass Antifade Mountant (Invitrogen). Confocal images were acquired using a Carl Zeiss LSM 800 confocal microscope.

### Cell viability assay

Huh7 cells were treated with 17-AAG of the indicated concentrations for 24 h, followed by the detection of cell viability using CellTiter-Glo® Luminescent Cell Viability Assay kit (Promega). The 50% cytotoxic concentration (CC_50_) was determined based on the results of three independent experiments.

### Statistical analysis

Student’s *t*-test was used for data analysis (GraphPad Prism 7.0). A *p* ≤ 0.05 was considered statistically significant.

## Results

### Hsp90 is required for MERS-CoV replication

**Figure 1.**
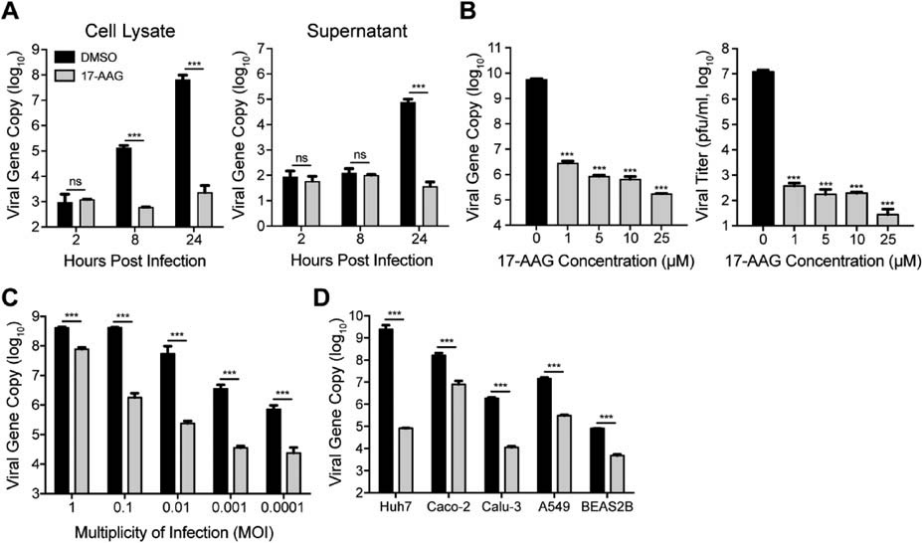
Hsp90 is required for MERS-CoV replication. (A) Huh7 cells treated with 10 µM 17-AAG or DMSO in triplicate were inoculated with MERS-CoV at a MOI of 0.01. At the indicated hours post infection, cell lysate and supernatant were collected for the viral gene copy detection. (B) Huh7 cells infected at a MOI of 0.01 were treated with various concentrations of 17-AAG. Cell-free culture media were harvested at 24 hpi for viral load quantification and plaque assay. (C) Huh7 cells treated with 10 µM 17-AAG were inoculated with MERS-CoV at the indicated MOIs. Cell-free culture media were harvested at 24 hpi for viral load quantification. (D) The indicated cells treated with 10 µM 17-AAG were inoculated with MERS-CoV at a MOI of 0.01. Cell-free culture media were harvested at 24 hpi for viral load quantification. Results present mean and SD of one representative experiment independently repeated three times. Student’s *t* test was used for data analysis. ***p ≤ 0.001.

To assess the role of Hsp90 in MERS-CoV replication, we tested the effect of an Hsp90 inhibitor, 17-AAG, in MERS-CoV-infected Huh7 cells, and monitored viral propagation. 17-AAG inactivates Hsp90 upon binding to the N-terminal ATP/ADP active site [23]. As shown in Figure 1, the intracellular viral loads were significantly lower in 17-AAG treated cells (cell lysate) than DMSO-treated cells (Figure 1(A)). Viral load in the culture medium (supernatant) decreased by 3 log units after 17-AAG treatment (Figure 1(A)). We performed additional experiments to verify the inhibitory effect of 17-AAG. 17-AAG treatment significantly reduced viral loads in the culture medium in a dose-dependent manner (Figure 1(B)). A remarkable viral reduction was verified by the viral titration which quantified the amounts of infectious virions (Figure 1(B)).

We further evaluated the inhibitory effect of 17-AAG on MERS-CoV replication at different MOIs ranging from 0.0001 to 1. Even after a high MOI (0.1 and 1 MOI) inoculation, significantly decreased viral loads of 1–2 log units were observed in the culture medium of 17-AAG-treated cells (Figure 1(C)). We then assessed the effect of 17-AAG in multiple human cell lines. 17-AAG treatment resulted in a general reduction of vial growth in all the tested cell lines including Caco-2, Calu-3, A549 and BEAS-2B (Figure 1(D)). To exclude the possibility that 17-AAG-mediated viral inhibition is related to the cytotoxicity, we measured its 50% cytotoxic concentration (CC_50_). As shown in Supplementary Figure 1, the CC_50_ was 50 µM in Huh7 cells. Except for a maximum concentration of 25 µM used in dose-dependency assay (Figure 1(B)), a uniform concentration of 10 µM 17-AAG was used for all the experiments in this study. Thus, 17-AAG induced viral inhibition is not related to any cytotoxicity. Collectively, the results suggested that Hsp90 is required for MERS-CoV propagation.

### Hsp90 inhibitor suppressed MERS-CoV replication in human intestinal organoids

**Figure 2.**
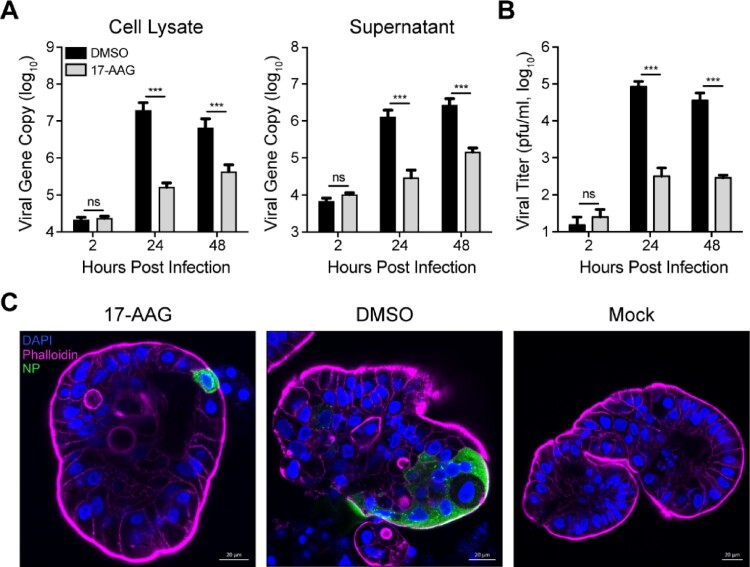
Hsp90 inhibition reduced MERS-CoV replication in intestinal organoids. The differentiated intestinal organoids treated with 10 µM 17-AAG or DMSO in triplicate were inoculated with MERS-CoV at an estimated MOI of 0.1. At indicated hours post infection, the organoids were applied to viral load detection, cell-free Matrigel and culture medium were applied to viral load detection (A) and viral titration (B). Results present mean and SD of one representative experiment independently repeated three times. Student’s *t* test was used for data analysis. ***p ≤ 0.001. (C) The infected or mock-infected organoids were fixed at 24 hpi, followed by immunofluorescence staining of MERS-CoV NP (green) and confocal imaging. Nuclei and cellular actin filaments are counterstained with DAPI (blue) and Phalloidin-647 (purple) respectively. Scale bar, 20 μm.

We previously demonstrated that human intestinal organoids are highly susceptible to MERS-CoV and sustain robust viral replication [19]. Human intestinal organoids are generated from LGR5+ adult stem cells in normal human intestines. The differentiated intestinal organoids possess all the mature epithelial cell types in human intestine and can morphologically and functionally simulate human intestinal epithelium to a near-physiological level [20,24]. Herein, the effect of Hsp90 inhibition on MERS-CoV replication was evaluated in the physiological-relevant human intestinal organoids. As shown in Figure 2(A), 17-AAG treatment significantly reduced viral loads in the organoids and in the culture medium. In the presence of 17-AAG, the production of infectious virions from the infected organoids generally ceased from 24 hpi (Figure 2(B)), indicating Hsp90 inhibition abolished viral replication remarkably in the organoids. Immunostaining of virus-infected cells and confocal imaging revealed only a few virus-positive (NP+) cells in the 17-AAG treated organoids. In contrast, abundant virus-infected cells were clearly discernible in the mock-treated organoids (Figure 2(C)).

### Genetic depletion of Hsp90β suppressed MERS-CoV replication and abolished virus spread

**Figure 3.**
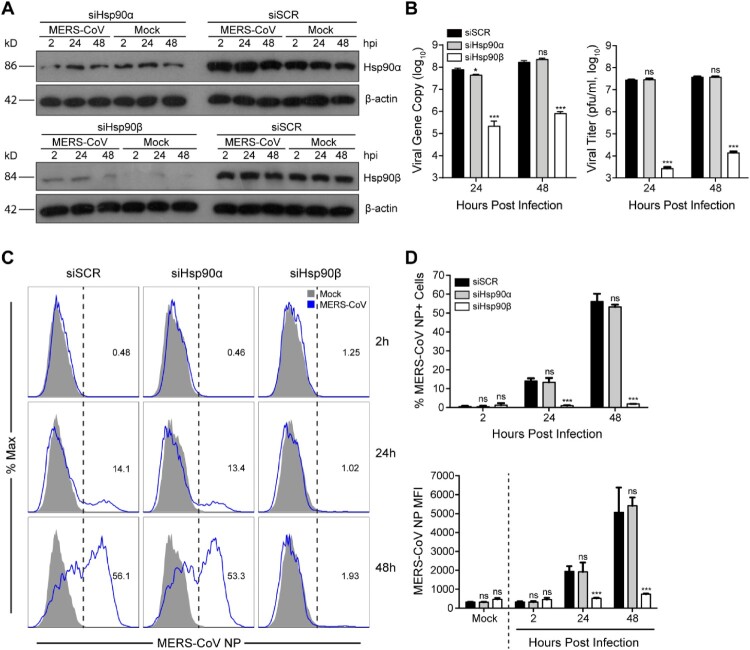
Genetic depletion of Hsp90β suppressed MERS-CoV replication and abolished virus spread. At 24 h after transfection of Hsp90α, Hsp90β or scrambled siRNA in triplicate, A549 cells were infected with MERS-CoV at a MOI of 0.1. (A) The expression levels of Hsp90α and Hsp90β in both infected and mock-infected cells were detected by Western blot. (B) At the indicated hours post infection, cell-free culture media were applied to viral load detection and viral titration. (C, D) The infected or mock-infected cells were fixed at the indicated hours post infection and labelled for flow cytometry. (C) Histograms show the infection rates of one representative experiment. (D) Results present mean and SD of infection rate and MFI of one representative experiment independently repeated three times. Student’s *t* test was used for data analysis. ***p ≤ 0.001, *p ≤ 0.05.

As aforementioned, Hsp90 has two cytosolic isoforms, Hsp90α and Hsp90β. We evaluated the individual role of two Hsp90 isoforms in MERS-CoV replication via genetic depletion. At 24 h after siRNA transfection, we inoculated MERS-CoV in the transfected cells and monitored viral growth. As shown in Figure 3(A), compared to scrambled siRNA (siSCR) transfected cells, Hsp90α and Hsp90β were depleted significantly after transfection of the respective siRNA oligos. Moreover, the depletion of both isoforms underwent minimal change during MERS-CoV propagation in Hsp90α or Hsp90β defective cells. Of note, Hsp90β depletion profoundly reduced viral growth as shown by both viral load quantification and viral titration, whereas Hsp90α depletion showed negligible effect (Figure 3(B)). Similar to 17-AAG treatment, Hsp90β depletion led to a dramatically reduced production of infectious virions of more than 4 log units at 24 h post infection. Again, we verified that temporal depletion of Hsp90α or Hsp90β had minimal impact on cell viability (Supplementary Figure 2), excluding the possibility of compromised cell viability contributable to the viral inhibition induced by Hsp90β depletion. A significant and specific reduction of MERS-CoV replication in Hsp90β depleted cells, but not Hsp90α-depleted cells, was also verified in primary human embryonic lung fibroblast (HELF) cells (Supplementary Figure 3).

To further validate the involvement of Hsp90β in MERS-CoV propagation, we monitored virus spread by flow cytometry after genetic depletion. In scrambled siRNA transfected cells, the percentage of virus-infected (NP+) cells increased from 0.48% at 2 hpi to 56.1% at 48 hpi, suggesting a productive infection. The cells with defective Hsp90α showed similar kinetics of virus spread (Figure 3(C,D)). In stark contrast to the active virus spread in Hsp90α-depleted cells and control cells, the infection rate in Hsp90β-depleted cells remained unchanged at baseline level over time, indicating a remarkably restricted viral growth (Figure 3(C,D)). The abundance of viral NP in the infected cells, as measured by mean fluorescent intensity (MFI), showed a pattern similar to the infection rate (Figure 3(D)). Collectively, Hsp90β genetic depletion significantly abolished viral growth and spread.

### Hsp90β interacts with MERS-CoV NP to maintain its stability

**Figure 4.**
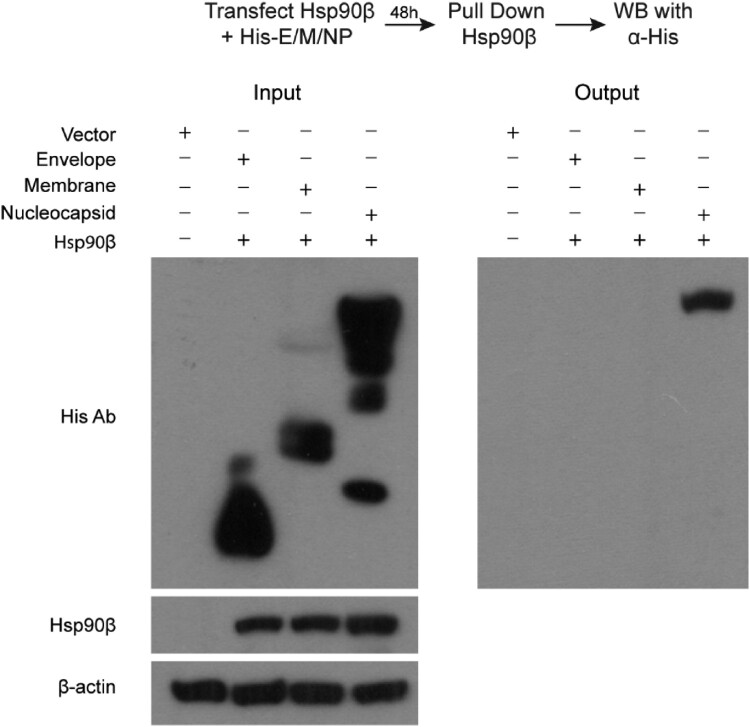
Co-IP assay identified MERS-CoV NP as an Hsp90β client protein. 293T cells were transfected with Hsp90β expression plasmid and His-tag plasmid expressing the indicated MERS-CoV structural proteins or blank vector. Cell lysates of the transfectants were applied to verify the expression of His-tagged viral proteins and Hsp90β. The cell lysates (input) were used for immunoprecipitation with an α-Hsp90β antibody; the co-precipitated partner (output) was detected by Western blot using an α-His antibody.

We proceeded to identify the viral partner(s), specifically MERS-CoV structural proteins, interacting with Hsp90β by co-immunoprecipitation (Co-IP) assay, based on the extensive documentation of Hsp90β interaction with viral structural proteins of many viruses [16]. To this end, 293T cells were co-transfected with an Hsp90β expression plasmid, and a His-tagged plasmid expressing MERS-CoV envelope, membrane, or nucleocapsid (NP) or the blank vector (Vector, Figure 4). At 48 h post transfection, cells were harvested and applied to immunoprecipitation using an α-Hsp90β antibody. The coprecipitated partners with Hsp90β were then detected by Western blot using an α-His antibody. MERS-CoV structural proteins, i.e. envelope, membrane and nucleocapsid, as well as Hsp90β were expressed properly after the pairwise co-transfection (Figure 4). After immunoprecipitation with the α-Hsp90β, MERS-CoV nucleocapsid, but not envelop, membrane protein or any His-tagged protein, was detected in the immunoprecipitated complexes (Figure 4). A similar Co-IP was performed after co-transfection of Hsp90β and Spike protein expression plasmid. The α-Hsp90β antibody was unable to pull down the overexpressed Spike protein (Supplementary Figure 4). These results suggest that among four MERS-CoV structural proteins, NP specially interacts with Hsp90β.

**Figure 5.**
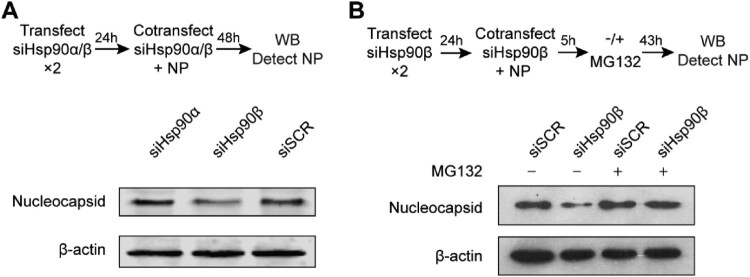
Hsp90β is required to maintain NP stability. (A & B) At 24 h post twice transfection of Hsp90α, Hsp90β or scrambled siRNA, NP plasmid was co-transfected with the respective siRNA. (A) The transfected cells were harvested at 48 h after co-transfection and applied to Western blot to detect the NP expression. (B) At 5 h post co-transfection of NP plasmid and Hsp90β or scrambled siRNA, the cells were incubated in the presence of absence or 1.25 µM MG132 for 43 h and then applied to NP detection by Western blot.

Given the interaction demonstrated above, we further evaluated the role of Hsp90β on MERS-CoV nucleocapsid (NP). To this end, one day after twice transfection of siHsp90α, siHsp90β or siSCR, the cells were co-transfected with NP plasmid and respective siRNAs and incubated for 48 h (Figure 5(A)). The transfected cells were harvested for detection of NP by Western blot. Compared to the cells transfected with siSCR, Hsp90β depletion considerably diminished the abundance of NP protein. Similarly, Hsp90α knockdown showed negligible effect (Figure 5(A)).

Molecular chaperones including Hsp90β interact with client proteins to promote protein folding, maturation and trafficking. In the context of the defective Hsp90, misfolded client proteins commonly undergo autophagic or proteasomal degradation [25]. We inspected whether proteasomal degradation is operational in Hsp90β depletion mediated NP reduction. At five hours post co-transfection of Hsp90β siRNA and NP plasmid as mentioned above, cells were incubated in the presence or absence of proteasome inhibitor MG132 for 43 h and then applied to Western blot. As shown in Figure 5(B), NP reduction induced by Hsp90β depletion was rescued when proteasomal degradation was abrogated by MG132 (lane 4 versus lane 2). Collectively, we identified MERS-CoV NP as an Hsp90β substrate. Hsp90β is required to maintain the stability of NP since Hsp90β depletion diminished NP protein. In the context of defective Hsp90β, MERS-CoV NP is prone to proteasomal degradation.

### Hsp90 is required for replication of divergent human coronaviruses

**Figure 6.**
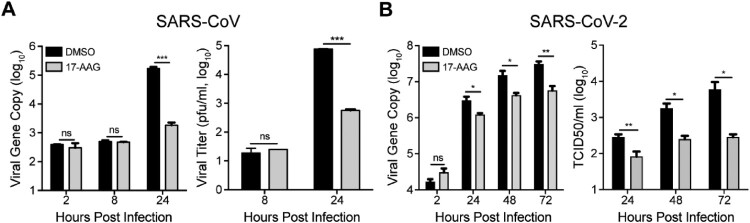
Hsp90 inhibitor suppressed SARS-CoV and SARS-CoV-2 replication. (A & B) Huh7 cells treated with 10 µM 17-AAG or DMSO in triplicate were inoculated with SARS-CoV or SARS-CoV-2. At the indicated hours post infection, cell-free culture media were collected for viral gene copy quantification and viral titration by plaque assay. Student’s *t* test was used for data analysis. ***p ≤ 0.001, **p ≤ 0.01, *p ≤ 0.05.

We next assessed whether Hsp90 is involved in viral growth of SARS-CoV and SARS-CoV-2, the other two highly pathogenic human coronaviruses. As shown in Figure 6(A), viral load in culture media was 2 log units lower in 17-AAG-treated Huh7 cells than DMSO-treated cells at 24 h post infection of SARS-CoV. A significantly decreased viral titer of more than 2 log units was also observed in the supernatant of 17-AAG treated cells. The role of Hsp90 was also tested in viral growth of SARS-CoV-2 in Huh7 cells. Similarly, 17-AAG treatment resulted in a significant reduction in virus production as shown by both viral load quantification and viral titration (Figure 6(B)). Thus, Hsp90 is involved in the replication of diverse human coronaviruses.

## Discussion

Hsp90 proteins, a family of abundantly expressed and highly conserved molecular chaperones, are the key regulator of proteostasis under physiological and stress conditions in eukaryotic cells [13,26]. Upon viral infection, large amounts of viral proteins are produced within a short period of time; the abundantly produced viral proteins reply on cellular chaperone mediated proteostasis for their structural and functional integrity. Hsp90 proteins are involved in folding, maturation and stabilization of many viral proteins, thus are almost universal required for replication of a variety of DNA and RNA viruses [17,27]. To assess the potential involvement of Hsp90 in the virogenesis of MERS-CoV, we tested the effect of an Hsp90 inhibitor 17-AAG on viral propagation. Hsp90 inhibitor-induced viral reduction occurred in a dose-dependent manner and in multiple permissive cell lines (Figure 1(B,D)). Human intestinal organoids morphologically and functionally simulate the native intestinal epithelium to a near-physiological level. We verified the involvement of Hsp90 for MERS-CoV replication in these physiological-relevant human intestinal organoids (Figure 2).

We moved on to resolve the specific effect of two cytosolic isoforms, Hsp90α and Hsp90β, on viral growth via genetic depletion. While Hsp90α depletion showed minimal effect on viral growth, Hsp90β depletion resulted in a dramatically decreased viral titer of more than 4 log units (Figure 3(B)). The dependency on Hsp90β for MERS-CoV replication, rather than the counterpart isoform Hsp90α, was recapitulated in the kinetics of virus spread as shown by flow cytometry (Figure 3(C,D)). Hsp90β depletion basically contained virus spread within the initial baseline level overtime. In addition, we note that, in both pharmacological inhibition of Hsp90 and genetic depletion of Hsp90β, the reduction of viral titer is invariably more prominent than that of viral gene copy number (Figure 1(B), Figure 2(A) and Figure 3(B)), indicating that large amounts of MERS-CoV defective virions were generated in both settings.

Given the dependency on Hsp90β for MERS-CoV propagation, we proceeded to probe the molecular mechanism. A Co-IP assay was performed to pinpoint NP as an Hsp90β client protein among four structural proteins of MERS-CoV (Figure 4). We demonstrate a direct interaction of Hsp90β with NP (Figure 4), and dependency of Hsp90β for the protein stability of NP. Upon Hsp90β depletion, NP is more prone to proteasomal degradation (Figure 5). Hsp90β interaction with MERS-CoV NP would be verified alternatively by optical technique such as bio-layer interferometry or surface plasmon resonance [28,29], which can determine biomolecular interactions quickly and accurately. As a multifunctional structural protein, coronavirus NP forms complex with genomic RNA, interacts with other structural proteins during virus assembly and virus budding, and plays a critical role in viral genome transcription and replication [30]. The functional complexity and structural requirement render NP highly dependent on chaperon system for proper folding, stability and divergent conformation change during virus life cycle. In this study, our data coherently demonstrate that Hsp90β functions to maintain the stability and/or functional integrity of MERS-CoV NP. Accordingly, the compromised NP in the context of inactive or defective Hsp90 leads to a significantly diminished viral propagation in cell lines and human intestinal organoids. Herein, we only examined the potential interaction of MERS-CoV structural proteins with Hsp90β, it definitely warrants further investigation to explore whether Hsp90 is required for the integrity of non-structural proteins.

COVID-19 pandemic has become a social-economic crisis worldwide. Based on an *in silico* prediction, Hsp90 inhibitors could be used to treat COVID-19 [31]. Herein, we provide the experimental evidence that 17-AAG potently suppressed the replication of SARS-CoV-2 and SARS-CoV (Figure 6), which highlights the potential of targeting Hsp90 as a promising therapeutic strategy against SARS-CoV-2. It is believed that Hsp90 may recognize a metastable structural element in client proteins rather than a primary amino acid motif [17]. Thus, Hsp90 inhibitor-mediated suppression of SARS-CoV-2 and SARS-CoV is readily expected, although the amino acid homology of MERS-CoV NP with SARS-CoV-2 NP and SARS-CoV NP is around 50%. Nevertheless, the viral target(s) of Hsp90 in SARS-CoV-2 and SARS-CoV definitely requires further investigation.

Similar to the virus-infected cells, highly proliferative cancer cells show a higher dependency on cellular chaperones than normal cells to prevent the toxic effects of intracellular protein misfolding and aggregation. As such, several pharmacological Hsp90 inhibitors have been developed, some of which have been in advanced clinical trials for cancer treatment [32]. These inhibitors could be repurposed as a novel class of antivirals to treat COVID-19. Up to now, there is no approved antiviral treatment for human coronavirus infections. As far as we know, 17-AAG-mediated inhibition is more potent than most reported anti-coronaviruses agents [33,34], if not all. Our study demonstrated the essential role of Hsp90β for replication of human coronaviruses. Thus, temporary inhibition of Hsp90 may represent a promising therapeutic strategy against human coronavirus infections.

## Supplementary Material

Supplementary_material_Nov_5.docx
