# Supplementary material for: Human coronavirus dependency on host heat shock protein 90 reveals an antiviral target
Source: Emerg Microbes Infect. 2020 Dec 17;9(1):2663–72. doi: 10.1080/22221751.2020.1850183 (PMC7751432; doi:10.1080/22221751.2020.1850183)

**Supplementary Figure Legends**

**Supplementary Figure 1. CC_50_ of 17-AAG in Huh7 cells.** Huh7 were treated with 17-AAG of the indicated concentrations in triplicate and incubated for 24 hours, followed with a cell viability assay. Results present mean and SD of three independent experiments.

**Supplementary Figure 2. The intact cell viability after Hsp90 depletion.** At 24 hours after the third transfection of Hsp90α, or Hsp90β or scrambled siRNA, the cell viability of A549 was detected. Results present mean and SD of three independent experiments.

**Supplementary Figure 3. Genetic depletion of Hsp90β suppressed MERS-CoV replication in HELF cells.** At 24 hours post the transfection of Hsp90α, Hsp90β or scrambled siRNA, HELF cells were infected with MERS-CoV at a MOI of 0.1. At the indicated hours post infection, culture media were applied to viral titration. Data show mean and SD of one representative experiment independently repeated three times. Student’s *t* test was used for data analysis. ***p ≤ 0.001, *p ≤ 0.05.

**Supplementary Figure 4.** 293T cells were transfected with Hsp90β expression plasmid and a plasmid expressing MERS-CoV spike protein or blank vector. Cell lysates of the transfectants were applied to verify the expression of spike and Hsp90β. The cell lysates (input) were used for immunoprecipitation with an α-Hsp90β antibody; the co-precipitated partner (output) was detected by Western blot using and an α-spike antibody.

Supplementary Figure 1


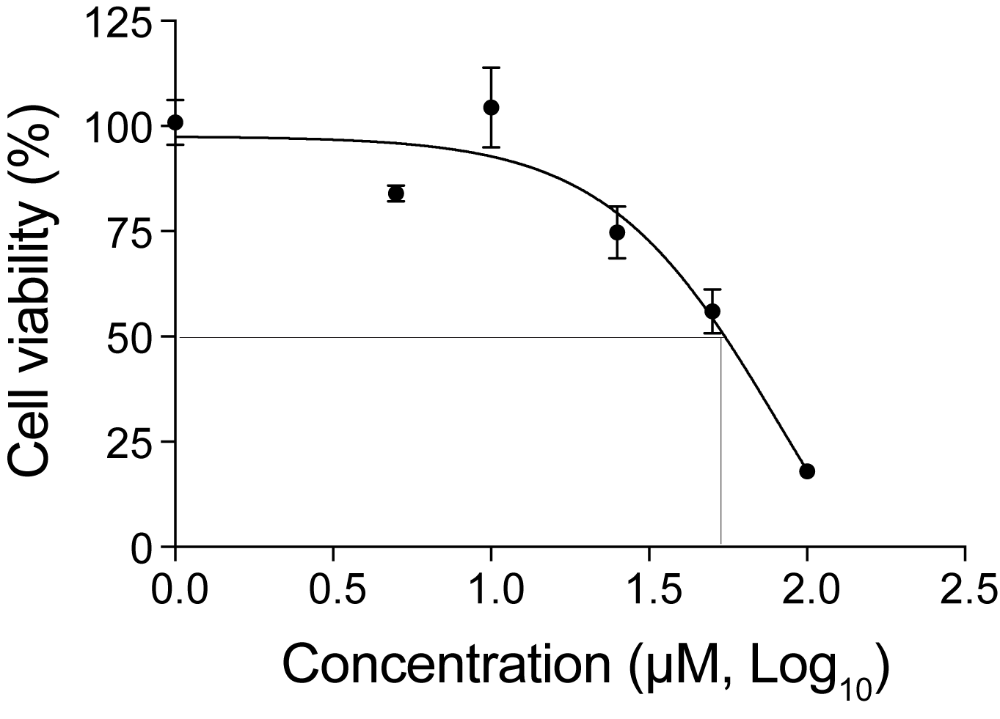


Supplementary Figure 2


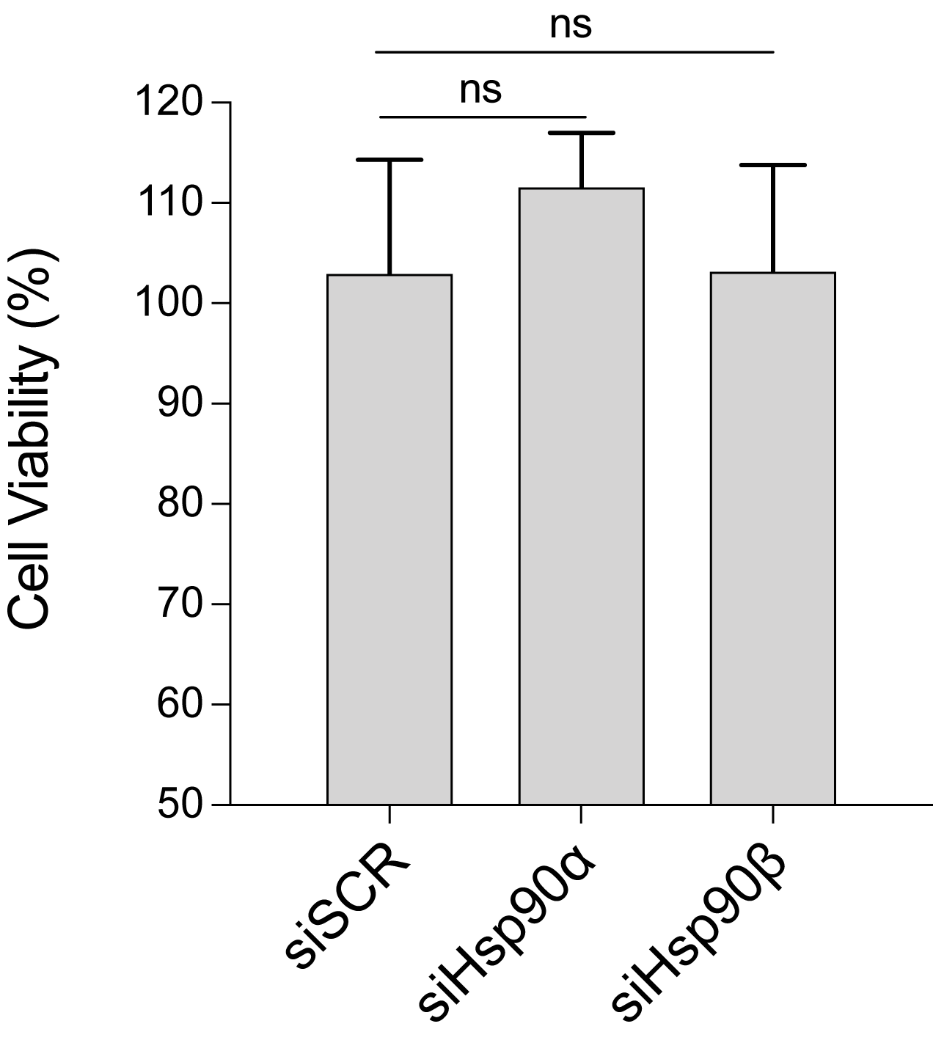


Supplementary Figure 3


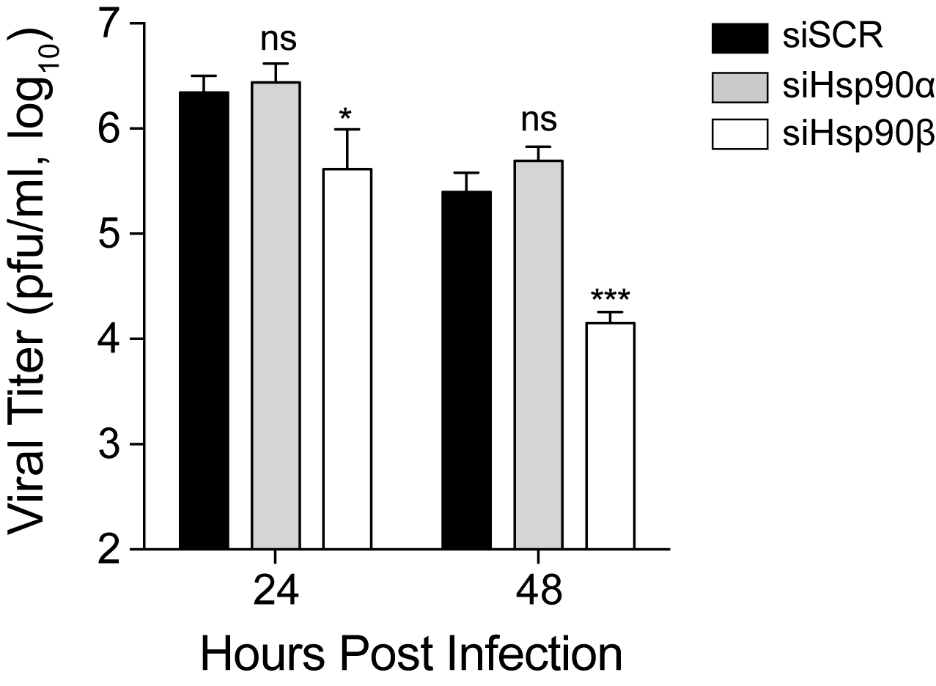


Supplementary Figure 4


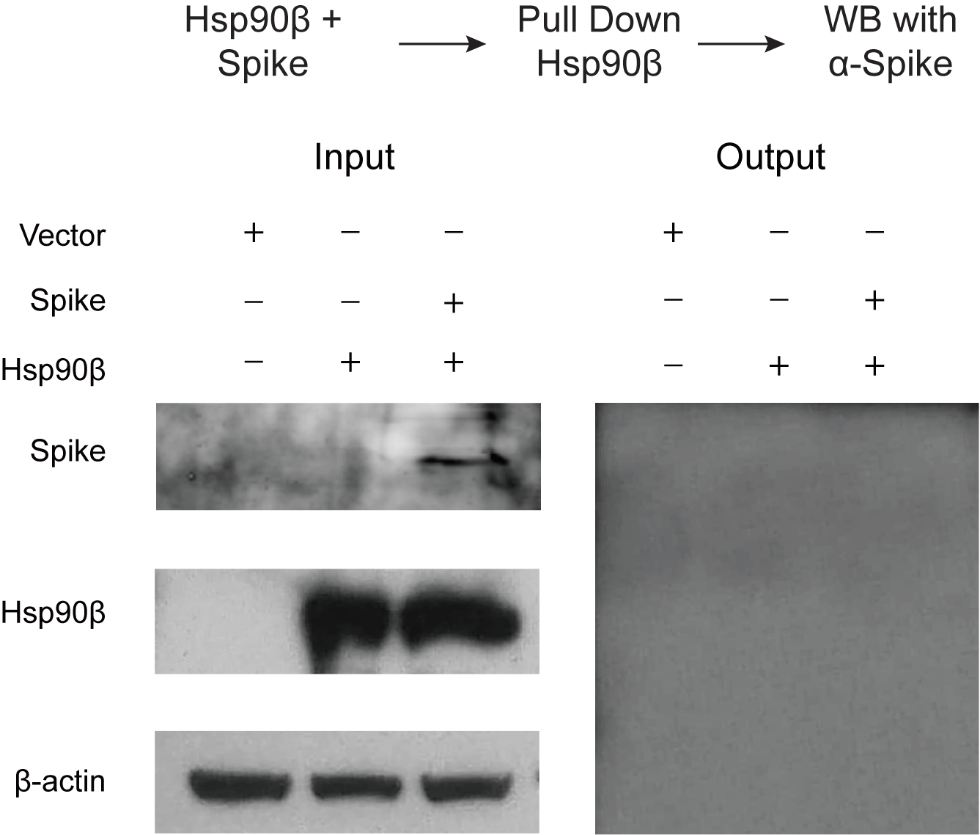

Supplement: Supplementary_material_Nov_5.docx [file TEMI_A_1850183_SM9678.docx]
